# Epitopes for a 2019-nCoV vaccine

**DOI:** 10.1038/s41423-020-0377-z

**Published:** 2020-02-24

**Authors:** Guglielmo Lucchese

**Affiliations:** Universitätsmedizin Greifswald, Department of Neurology, 17475 Greifswald, Germany

**Keywords:** Infectious diseases, Vaccines

After causing an initial cluster of Pneumonia in Wuhan City, Hubei Province, the 2019-nCoV has quickly spread through South East Asia and within a few weeks to Europe, Africa, and America. Initial estimates suggested a mortality rate of 2% and that ~18% of the cases show severe symptoms, although such estimates are still subject to rapid changes (https://www.who.int/news-room/detail/30-01-2020-statement-on-the-second-meeting-of-the-international-health-regulations-(2005)-emergency-committee-regarding-the-outbreak-of-novel-coronavirus-(2019-ncov)).^1–3^

To facilitate the swift development of a candidate vaccine for 2019-nCoV we compared here the viral and the human proteomes, searching for pentapeptides that are unique to the pathogen. We followed the rationale that non-self sequences are highly immunogenic and uniquely viral epitopes should improve safety and efficacy by minimizing the risk for cross-reactions and increasing anti-viral specificity.^4–6^ The analysis was conducted on the entire viral proteome but primarily focused on the surface spike glycoprotein (id = “QHD43416.1) because immune response against it is highly likely to exert a neutralizing effect.^2^

The entire amino acid (aa) sequence of the 2019-nCoV was retrieved from https://www.ncbi.nlm.nih.gov/nuccore/MN908947 and dissected into pentapeptides overlapped by four residues for a total of *n* = 9661. Then, each pentamer was analyzed for occurrences in the human proteome using the Peptide Match program (https://research.bioinformatics.udel.edu/peptidematch/index.jsp).^7^

It resulted that *n* = 933 viral pentapeptides are absent in the human proteome, and therefore foreign to the human immune system (Table S1). Among these non-self pentapeptides, *n* = 107 are embedded in the viral surface glycoprotein (spike protein) that mediates binding to the human ACE2 and cellular entry.^2^

The recommended oligopeptides for a multi-epitope 2109-nCoV-vaccine are presented in Table 1, Panel a. They can be rapidly tested in animal models for immunogenicity and safety in order to timely develop a vaccine for preventing uncontrolled spreading of the novel coronavirus.

**Table 1:** (a) Oligopeptides (*n* = 73) of the spike protein absent in the human proteome to be tested for a potential vaccine. Contiguous pentapeptides with a four residue overlap were considered as a single, longer oligopeptidic sequence; the length of each of these oligopeptides was dictated by the extension of the overlap. Oligopeptides from epitopes in panel b are in bold. (b) Experimentally validated epitopes (*n* = 66) containing at least one of the 107 pentapeptides (capitalized) of the spike protein that are absent in the human proteome

(a)	**RGVYYPD**K, NVTWFHA, FHAIH, PFNDG, IRGWIF, IFGTT, VCEFQFC, CNDPF, VYYHK, NNKSW, NKSWM, WMESEF, YSSAN, **CTFEY**, GNFKN, **GYFKI**, IYSKHT, PIGIN, GWTAG, AYYVG, NENGT, SETKC, **GIYQT**, VYAWNR, **CVADY**, **STFKC**, **FKCYGVS**, TNVYA, **IADYN**, **DYNYKL**, VIAWN, AWNSNN, STPCN, PCNGV, GFNCYF, QSYGF, VKNKC, NKCVN, **CVNFN**, CTEVP, **IGAEH**, YQTQTN, I**AYTMS**, **TSVDC**, DCTMY, T**MYICG**, **DSTEC**, **FCTQL**, PIKDF, QYGDCL, GDCLG, DLICAQKF, MIAQY, SGWTF, **WTFGA**, **FAMQM**, **MQMAYRF**, **RFNGI**, **MSECV**, **GYHLMS**, KNFTT, **PAICH**, NGTHWFVTQ, **TQRNF**, NFYEP, IGIVN, **NTVYD**, IKWPWYI, YIWLGF, **IAIVM**, **LCCMTS**, **MTSCC**, **CCKFD**	
(b)	**IEDB-ID-Number**	**Epitope**
	307	aalvsgtatagWTFGAg
	462	aatkMSECVlgqskrvd
	1460	agclIGAEHvdtsyecd
	3176	aMQMAYRF
	6011	canlllqygsFCTQLnralsgia
	6333	cgpklstdliknqCVNFNfngltgtgvltpsskrfqpfqqfg
	6334	cgpklstdliknqCVNFNfngltgtgvltpsskrfqpfqqfgrdvsdftd
	7066	csqnplaelkcsvksfeidkGIYQTsnfrvvpsgd
	7217	cttfddvqapnytqhtssmRGVYYPDeifr
	7383	CYGVSatklndlcfsnv
	8239	dfcgkGYHLMSfpqaap
	12417	eidkGIYQTsnfrvvps
	15903	ffSTFKCYGVSatklnd
	18161	fvfngtswfiTQRNFfs
	18515	gaalqipFAMQMAYRFn
	21464	gnliaprGYFKIrsgkssim
	22321	gsFCTQLn
	24978	htssmRGVYYPDeifrs
	25250	IADYNYKLpddfmgcvl
	25293	iaglIAIVMvtillccm
	25378	iapgqtgvIADYNYKLp
	25382	iaprGYFKIrngkssimrsdapigtcssecit
	29728	iywtivkpgdillinstgnliaprGYFKIrn
	30987	kGIYQTsn
	30988	kGIYQTsnfrvvpsgdvvrf
	31581	kkisnCVADYsvlynst
	31582	kkisnCVADYsvlynstf
	33305	ksfeidkGIYQTsnfrvv
	33358	ksivAYTMSlgadssia
	33874	kTSVDCnMYICGDSTEC
	36579	liknqCVNFNfngltgt
	36815	lkcsvksfeidkGIYQT
	36856	lkgacscgsCCKFDedd
	37758	llrstsqksivAYTMSl
	39023	lqygsFCTQLnralsgi
	41177	MAYRFNGIgvtqnvlye
	42999	mvtilLCCMTSCCsclk
	43145	nafnCTFEYisdafsld
	46379	nvfqtqagclIGAEHvd
	46822	PAICHegkayfpregvfvfngtswfitqrnffs
	47479	pFAMQMAYRFNGIgvtq
	49968	pvsmakTSVDCnMYICGds
	50058	pwyvwlgfiaglIAIVM
	53202	rasanlaatkMSECVlg
	54989	rnfttaPAICHegkayf
	58143	sgncdvvigiinNTVYD
	58730	sivAYTMSl
	61554	stdliknqCVNFNfn
	61598	stffSTFKCYGVSatkl
	62872	tagWTFGAgaalqipfa
	63309	tecanlllqygsFCTQL
	68971	vigiinNTVYDplqpel
	72205	VYYPDeifrsdtlyltqd
	74173	yicgDSTECanlllqyg
	75920	ysvlynstffSTFKCYG
	99918	CTFEYisdafsld
	100048	gaalqipFAMQMAYRF
	100230	ksivAYTMSlgadssiay
	100300	MAYRFNGIgvtqnvly
	100316	nafnCTFEYisdafsldv
	100537	swfiTQRNFfspqii
	100711	agclIGAEHvdtsyecdi
	129239	liaprGYFKIrsgkssi
	532052	gtswfiTQRNFfspq
	873061	mmcehiyytcvrTSVDCc
	874104	ytcvrTSVDCcmkgaep

*IEDB* Immune Epitope Database, *aa* amino acid

Three points need to be stressed.

First, short peptides that are foreign to the host immune system have been experimentally validated not only as positive immunomodulants (i.e., adjuvants) in conjunction to vaccines, but are also evidenced as providing direct protection against lethal viral infections, at least in animal models.^6^

Second, searching for the 107 human-foreign spike protein pentapeptides in the Immune Epitope Database (IEDB; www.iedb.org)^8^ yielded a list of *n* = 66 epitopes (Table 1, Panel b). The IEDB is a publicly available, curated epitope repository. The presence of a peptide sequence in the IEDB indicates that it has a recognized and experimentally proven immunologic relevance. These results provide experimental proof for the immunogenic potential of the non-self peptides identified in the present study through comparative *Homo sapiens*-coronavirus proteome analysis.

Third, an immune response induced by the spike protein oligopeptides that are absent in the human proteins would exert a neutralizing effect on the coronavirus, in light of the mounting evidence for the surface glycoprotein as a ligand for the human ACE2 in viral entry processes.^2^

## Supplementary information

Table S1
