# Supplementary material for: Epitopes for a 2019-nCoV vaccine
Source: Cell Mol Immunol. 2020 Feb 24;17(5):539–40. doi: 10.1038/s41423-020-0377-z (PMC7091830; doi:10.1038/s41423-020-0377-z)
Supplement: Supplementary file 1 — Table S1 [file 41423_2020_377_MOESM1_ESM.docx]

**Table S1. Distribution along the 2019-nCoV proteome of pentapeptides unique to the virus and absent in the human proteome.**

| **ORF1ab polyprotein / id= QHD43415.1 (n=709):** |
| --- |
| PGFNE, FNEKT, THVQL, QPYVF, YVFIK, HVMVE, HSYGA, TDPYE, QENWN, ENWNT, NTKHS, TKHSS, DNNFC, YPLEC, CIKDL, FIDTK, KRGVY, RGVYC, EIAWY, AWYTE, QTPFE, ECPNF, NFVFP, RSVYP, SPNEC, NQMCL, MKCDH, WQTGD, EFCGT, ATTCG, TTCGY, VKIYC, CHNSE, AEYHN, EYHNE, YHNES, HNESG, YVGCH, VGCHN, GCHNK, KCAYW, CAYWV, ANIGC, GCNHT, NHTGV, KGAWN, AWNIG, WNIGE, WLTNI, WEIVK, ACEIV, QTFFK, CADSI, YRKCV, RKCVK, PVCIN, KYCAL, YCALA, LAPNM, PNMMV, MVTNN, QGYKS, YKSVN, KCSAY, NEFAC, LDEWS, DEWSM, WSMAT, MATYY, YYLFD, SHMYC, YCSFY, CSFYP, YPPDE, TQYEY, QYEYG, WLDDD, TDNVY, YIKNA, ESDDY, KHCLH, IAEIP, TKFLT, VIPTK, GTTEM, VPTDN, PTDNY, DNYIT, NYITT, TTYPG, WNLRE, LMPVC, MPVCV, YGARF, FYFYT, VTMPL, YVTHG, ARYMR, TAYNG, HFIET, GSYKD, KDWSY, WSYSG, INLHT, DMSMT, MSMTY, MTYGQ, YGQQF, IKPHN, KPHNS, PHNSH, HNSHE, EAFEY, RYMSA, HTKKW, IKWAD, WADNN, LAYCN, AYCNK, YCNKT, CNKTV, YLFQH, FQHAN, HANLD, VVCKT, AVMYM, CTCGK, CGKQA, PFVMM, MMSAP, PAQYE, HGTFT, TFTCA, EYTGN, TGNYQ, NYQCG, QCGHY, GHYKH, HYKHI, LYCID, YCIDG, TDVFY, VCTEI, DNSYF, EQPID, NQPYP, QPYPN, FKFVC, DYKHY, YKHYT, YTPSF, HKPIV, PIVWH, VWHVN, WHVNN, HVNNA, KPNTW, PNTWC, NTWCI, TWCIR, WCIRC, CIRCL, IRCLW, RCLWS, LWSTK, GMDNL, NPTIQ, VLECN, ECNVK, KPANN, HTDLM, WDTIA, NYAKP, YAKPF, RCLNR, CTNYM, TNYMP, NYMPY, YMPYF, MPYFF, PYFFT, ASFNY, IIIWF, GMPSY, MPSYC, PSYCT, YCTGY, TGYRE, TYCTG, SFKWD, AEWFL, WFLAY, IMQLF, NSWLM, SWLMW, WLMWL, LMWLI, MWLII, AMVRM, MVRMY, RMYIF, MYIFF, SFYYV, FYYVW, YYVWK, STCMM, CMMCY, MMCYK, MCYKR, TRVEC, CTTIV, KLHNW, LHNWN, HNWNC, NWNCV, WNCVN, CVNCD, VNCDT, NCDTF, CDTFC, DTFCA, KRPIN, TYERH, NVIVF, ASVYY, YSQLM, MFDAY, DVVEC, NNYML, MLTYN, YNKVE, ENMTP, RHINA, KSHNI, FMSLS, KIVNN, PVHVM, VMSKH, KHTDF, TRDIA, DTCFA, CFANK, HADFD, DFDTW, FDTWF, TWFSQ, YTNDK, DKACP, CYTPS, AAECT, PYCYD, YCYDT, CYDTN, YCRHG, TCERS, RWVLN, LNNDY, PGVFC, LTNMF, CLAYY, AYYFM, YYFMR, YFMRF, FMRFR, EYSHV, SHVVA, VAFNT, LTPVY, YSVIY, AHIQW, HIQWM, QWMVM, WMVMF, MVMFT, VPFWI, PFWIT, TIAYI, IAYII, AYIIC, YIICI, TKHFY, HFYWF, FYWFF, YWFFS, WFFSN, EAACC, VEGCM, QVTCG, YCPRH, CPRHV, KSNHN, GHSMQ, SMQNC, MQNCV, QNCVL, YKFVR, QCAMR, CAMRP, AMRPN, MRPNF, FTIKG, DYDCV, CVSFC, FCYMH, CYMHH, YMHHM, MHHME, HHMEL, AWLYA, NGDRW, DFNLV, MKYNY, TQDHV, QTGIA, VLDMC, NGMNG, DEFTP, EFTPF, FDVVR, GVTFQ, GTHHW, THHWL, QSTQW, QWSLF, WSLFF, PFAMG, FAMMF, AMMFV, VKHKH, KHKHA, NMVYM, SWVMR, RIMTW, IMTWL, TWLDM, DMVDT, MVDTS, DCVMY, MYASA, RTVYD, VWTLM, WTLMN, YKVYY, KVYYG, VYYGN, AISMW, ISMWA, SMWAL, MWALI, TTVMF, GIVFM, VFMCV, FMCVE, EYCPI, CPIFF, LQCIM, CIMLV, MLVYC, CTCYF, CYFGL, EFRYM, FRYMN, PCIKV, QSKMS, SKMSD, DVKCT, KCTSV, LWAQC, QLHND, ADQAM, DQAMT, AMTQM, VTSAM, AMQTM, INNAR, DYNTY, TYKNT, YKNTC, KNTCD, WEIQQ, ADSKI, ISMDN, MDNSP, PNLAW, NLAWP, QMSCA, MSCAA, QTACT, TACTD, NALAY, AYYNT, QDLKW, LKWAR, WARFP, EPPCR, PPCRF, PCRFV, YFIKG, LNRGM, PITNC, LCTHT, CCLYC, CLYCR, CRCHI, CHIDH, KGFCD, TCAND, VCGMW, CGMWK, RLTPC, PCGTG, FDIYN, DIYND, IYNDK, YNDKV, AGFAK, CCRFQ, VVKRH, FSNYQ, SNYQH, NYQHE, VAKHD, AKHDF, KHDFF, DFFKF, FFKFR, IDGDM, TKYTM, FDEGN, EGNCD, CDTLK, YNCCD, CCDDD, CDDDY, YFNKK, NKKDW, RVYAN, TVQFC, VQFCD, MRNAG, NGNWY, NWYDF, WYDFG, MPILT, ESHVD, HVDTD, YIKWD, IKWDL, YFKYW, FKYWD, YWDQT, WDQTY, DQTYH, LDDRC, DRCIL, RCILH, CILHC, CANFN, ANFNV, VSTGY, YHFRE, HNQDV, NQDVN, DPAMH, CFSVA, FYDFA, YDFAV, FFFAQ, NLPTM, PTMCD, TMCDI, MCDIR, KYFDC, YFDCY, DCYDG, CYDGG, INANQ, NANQV, FPFNK, PFNKW, FNKWG, NKWGK, KWGKA, YDSMS, YEDQD, TQMNL, NRART, TNRQF, IGTSK, KFYGG, FYGGW, WHNML, HNMLK, LMGWD, MGWDY, GWDYP, WDYPK, PKCDR, CDRAM, DRAMP, AMPNM, MLRIM, ARKHT, HTTCC, ECAQV, EMVMC, TANVN, YECLY, CLYRN, FSMMI, NSTYA, LYYQN, YYQNN, EAKCW, AKCWT, CWTET, HEFCS, FCSQH, SQHTM, HTMLV, QGDDY, YVYLP, MIERF, KHPNQ, NQEYA, VFHLY, GHMLD, SRYWE, WEPEF, MYTPH, YTPHT, FLCCK, CCKCC, CKCCY, CYDHV, VNPYV, NPYVC, GGMSY, YYCKS, YKNTC, KNTCV, NTCVG, ATCDW, TCDWT, CDWTN, DWTNA, NAGDY, LSYGI, SYGIA, RNYVF, QIGEY, YRGTT, HTVMP, PQEHY, YVRIT, SHFAI, HFAIG, RIVYT, YTACS, YLPID, PIDKC, RVECF, ECFDK, QYVFC, YVYIG, NSVCR, VCRLM, KTIGP, PDMFL, GTCRR, CPAEI, SAQCF, AQCFK, QCFKM, CFKMF, FKMFY, NPAWR, YDYVI, FTQTT, HSCNV, CNVNR, ILCIM, LCIMS, MSDRD, QAENV, FKDCS, VDTKF, DMTYR, MTYRR, MMGFK, GFKMN, FKMNY, KMNYQ, MNYQV, NYQVN, PNMFI, RAWIG, WIGFD, FDVEG, GCHAT, GDQFK, DQFKH, LPWNV, VFVLW, VKIGP, IGPER, CDRRA, RRATC, YACWH, ACWHH, CWHHS, WHHSI, HHSIG, IGFDY, GFDYV, FDYVY, NPFMI, PFMID, DVQQW, VQQWG, QQWGF, QWGFT, HGNAH, CDAIM, MTRCL, KRVDW, RVDWT, VDWTI, WTIEY, CRKVQ, KVQHM, FYSYA, YATHS, ATHSD, LFWNC, WNCNV, CNVDR, VDRYP, NSIVC, RFDTR, FYYSD, PCESH, TCITR, AVCRH, RHHAN, HANEY, AYNMM, YNMMI, WVYKQ, YNLWN, WNTFT, FDGQQ, NTVYT, ELWAK, WDYKR, MTDIA, VKTQF, EFKPR, YAFEH, HIVYG, DFIPM, KNYFI, SFMLW, FMLWC, MLWCK, LWCKD, WCKDG, CKDGH, VETFY, VAMPN, YKMQR, KMQRM, KGIMM, GIMMN, IMMNV, KYTQL, PYNMR, MRVIH, CATVH, TANKW, ANKWD, NKWDL, ISDMY, SDMYD, TYICG, GFIQQ, KITEH, EHSWN, HSWNA, YKLMG, GHFAW, HFAWW, FAWWT, AWWTA, WWTAF, LIGCN, IGCNY, GYVMH, YVMHA, MHANY, HANYI, YIFWR, IFWRN, WRNTN, TNPIQ, SLFDM, MSKFP, INDMI, NDMIL |
| **surface glycoprotein / id= QHD43416.1 (n. 107):** |
| RGVYY, GVYYP, VYYPD, YYPDK, NVTWF, VTWFH, TWFHA, FHAIH, PFNDG, IRGWI, RGWIF, IFGTT, VCEFQ, CEFQF, EFQFC, CNDPF, VYYHK, NNKSW, NKSWM, WMESE, MESEF, YSSAN, CTFEY, GNFKN, GYFKI, IYSKH, YSKHT, PIGIN, GWTAG, AYYVG, NENGT, SETKC, GIYQT, VYAWN, YAWNR, CVADY, STFKC, FKCYG, KCYGV, CYGVS, TNVYA, IADYN, DYNYK, YNYKL, VIAWN, AWNSN, WNSNN, STPCN, PCNGV, GFNCY, FNCYF, QSYGF, VKNKC, NKCVN, CVNFN, CTEVP, IGAEH, YQTQT, QTQTN, IAYTM, AYTMS, TSVDC, DCTMY, TMYIC, MYICG, DSTEC, FCTQL, PIKDF, QYGDC, YGDCL, GDCLG, DLICA, LICAQ, ICAQK, CAQKF, MIAQY, SGWTF, WTFGA, FAMQM, MQMAY, QMAYR, MAYRF, RFNGI, MSECV, GYHLM, YHLMS, KNFTT, PAICH, NGTHW, GTHWF, THWFV, HWFVT, WFVTQ, TQRNF, NFYEP, IGIVN, NTVYD, IKWPW, KWPWY, WPWYI, YIWLG, IWLGF, IAIVM, LCCMT, CCMTS, MTSCC, CCKFD |
| **ORF3a protein / id= QHD43417.1 (n. 29):** |
| LFMRI, FMRIF, PSDFV, PFGWL, GWLIV, FVTVY, INFVR, FVRII, IMRLW, WLCWK, LCWKC, WKCRS, YFLCW, FLCWH, LCWHT, CWHTN, WHTNC, HTNCY, TNCYD, CYDYC, YDYCI, YCIPY, CIPYN, EHDYQ, YQIGG, CVVLH, LYSTQ, HVQIH, YDEPT |
| **Envelope protein / id= QHD43418.1 (n=7):** |
| RLCAY, CAYCC, AYCCN, YCCNI, CCNIV, KPSFY, YVYSR |
| **Membrane glycoprotein / id= QHD43419.1 (n=17):** |
| MADSN, LEQWN, EQWNL, LTWIC, YRINW, RINWI, INWIT, GGIAI, SMWSF, MWSFN, NPETN, RIAGH, RCDIK, AAYSR, RYRIG, RIGNY, IGNYK |
| **ORF6 protein / id= QHD43420.1 (n=1):** |
| NKYSQ |
| **ORF7a protein / id= QHD43421.1 (n=6):** |
| LYHYQ, HYQEC, YQECV, GNSPF, QFAFA, FAFAC |
| **ORF8 protein / id= QHD43422.1 (n=14):** |
| FHQEC, QSCTQ, QHQPY, DPCPI, PCPIH, CPIHF, PIHFY, FYSKW, SKWYI, QYIDI, YTVSC, INCQE, EYHDV, YHDVR |
| **Nucleocapsid phosphoprotein / id= QHD43423.2 (n=37)** |
| QNQRN, NAPRI, TASWF, ASWFT, WFTAL, DDQIG, DQIGY, QIGYY, TRRIR, SPRWY, WYFYY, YYLGT, AGLPY, DGIIW, GIIWV, IIWVA, DHIGT, NPANN, PANNA, KGFYA, FYAEG, SKMSG, KAYNV, PEQTQ, QTQGN, QGTDY, TDYKH, YKHWP, KHWPQ, HWPQI, WPQIA, IAQFA, GMSRI, MSRIG, SRIGM, YKTFP, QQSMS |
| **ORF10 protein / id= QHI42199.1 (n=6):** |
| MGYIN, YINVF, NVFAF, LCRMN, CRMNS, MNSRN |
